# Difficulties in Adaptation of the Mother and Newborn via Cesarean Section versus Natural Birth—A Narrative Review

**DOI:** 10.3390/life13020300

**Published:** 2023-01-21

**Authors:** Vasile Valeriu Lupu, Ingrith Crenguta Miron, Anca Adam Raileanu, Iuliana Magdalena Starcea, Ancuta Lupu, Elena Tarca, Adriana Mocanu, Ana Maria Laura Buga, Valeriu Lupu, Silvia Fotea

**Affiliations:** 1 Pediatrics, “Grigore T. Popa” University of Medicine and Pharmacy, 700115 Iasi, Romania; 2 Department of Surgery II—Pediatric Surgery, “Grigore T. Popa” University of Medicine and Pharmacy, 700115 Iasi, Romania; 3 Pediatrics, Vaslui Emergency County Hospital, 730006 Vaslui, Romania; 4 Medical Department, Faculty of Medicine and Pharmacy, “Dunarea de Jos” University of Galati, 800008 Galati, Romania

**Keywords:** newborns, cesarean section, abnormalities in adaption

## Abstract

Birth is a physiological act that is part of the morpho-functional economy of the maternal body. Each stage in the act of birth has a predetermined pathway that is neurohormonally induced and morpho-functionally established through specific and characteristic adaptations. Like maternity, childbirth also has an important impact on the maternal body as a biological structure and psycho-emotional behavior. Cesarean section performed at the request of the mother with no medical underlying conditions besides the prolonged hospitalization risk can also cause breathing problems in children, delayed breastfeeding, and possible complications in a future pregnancy. Vaginal birth remains the path of choice for a physiological evolution pregnancy. Although erroneously considered safe and easy today, cesarean section delivery must remain an emergency procedure or a procedure recommended for pregnancies where birth is a risk to the mother and to the child, as cesarean section itself is a risk factor for negative outcomes for both mother and baby. This review summarizes the impact that both cesarean section and natural birth have on mother and newborn in their attempt to adapt to postpartum events and extrauterine life.

## 1. Introduction

When we talk about vaginal birth or cesarean birth, we have in mind childbirth, that is, the process by which the product of conception—the newborn—separates from the maternal environment and begins its life under completely new conditions. Physiologically, this passage is gradual and phased, equally including maternal participation and fetal adaptation in an absolutely physiological manner that characterizes labor for the human species [1,2]. The World Health Organization (WHO) defined normal birth as “spontaneous in onset, low-risk at the start of labor and remaining so throughout labor and delivery. The infant is born spontaneously in the vertex position between 37 and 42 completed weeks of pregnancy. After birth, mother and infant are in good condition” [3]. As for vaginal birth, it includes spontaneous vaginal delivery without labor-inducing drugs, induced vaginal delivery using drug, or other techniques to induce labor and assisted vaginal delivery that uses a specific instrument such as forceps or a vacuum device to extract the newborn. Both natural and induced vaginal delivery can be assisted. The term “natural childbirth” is used to describe a vaginal delivery without medication for pain or to start or speed up labor [4,5].

The aim of this review is to summarize the impact that both cesarean section and natural birth have on mother and newborn regarding their physical and emotional health in their attempt to adapt to postpartum period and childcare for the mother and to extrauterine life for the newborn.

## 2. Impact of Vaginal Birth on the Mother

**Figure 1:**
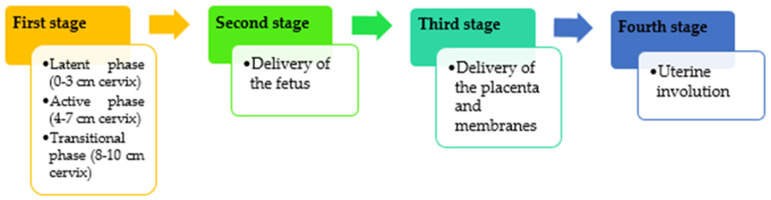
Labor stages.

To understand the adaptation effort to which the mother and newborn are subjected, a brief incursion into the physiology of birth with its two components is necessary. First, the maternal component prepares the act of childbirth through a series of morpho-functional changes facilitating the evolution of birth. Thus, labor, for example, is preceded by hormonal changes starting 3 to 4 weeks before the actual moment [6]. Labor initiation involves withdrawn functional progesterone and increased estrogen bioavailability, corticotrophin releasing hormone (CRH), and neuroendocrine mediators associated with an increased responsiveness of the myometrium to prostaglandins and oxytocin [7]. With a myometrial effect, in which the myometrium inhibitory hormones (such as progesterone of ovarian or placental origin) disappear, followed by the development of hormones that stimulate excitability, contractility, and development of the uterine muscles, such as oxytocin and estrogens [2,8]. Simultaneously, uterine contractions will gain consistency in intensity and frequency under the influence of excito-motor hormones and acetylcholine, leading, among other things, to progressive dilation of the cervix [8,9,10]. All these processes have a dynamic character, manifesting along four stages. The first stage of labor begins with mild, irregular uterine contractions in the latent phase that tend to become stronger and rhythmic. The active phase starts at 3–4 cm dilatation of the cervix and ends when full dilatation is achieved (10 cm). This active phase can take 4–8 h in nulliparous women with upper limits up to 20 h. In parous women, the median duration is about 2–5 h with an upper limit of up to 14 h [11,12]. The second stage, the expulsion, starts with full cervical dilatation and ends with the delivery of the fetus. The American College of Obstetricians and Gynecologists (ACOG) considers that if the second stage takes more than 3 h when regional anesthesia is associated in nulliparous women or 2 h in the absence of anesthesia, then this second stage is considered prolonged. The same statement applies for multiparous women if the second stage exceeds 2 h when given regional anesthesia or 1 h without [13]. The third stage starts after the complete delivery of the fetus and ends with completed delivery of the placenta and its attached membranes. The delivery of the placenta usually takes less than 10 min although the third stage of labor can last for 30 min. After 30 min, this stage is considered to be prolonged. The fourth stage includes the uterine involution that follows the delivery of the fetus and the placenta [7]. Both mother and newborn recover from the physical process of birth and also start bonding to each other [13,14] (Figure 1). 

Note that all these changes occur over time, and their actual performance in childbirth takes 8–12 h. Apart from the local pelvic-genital changes, there are changes and adaptation incurred by all organs, apparatuses, and systems of the maternal body. Thus, the rhythm and amplitude of breathing increases by about 20% (24–26 rpm and 600 mL of the current volume), heart rhythm increases to 80–85/min, AP increases by 10–20 mmHg, and cardiac output by 20–30% [15,16]; within the digestive system, there can be reflex vomiting, compression transit disorders, micturition disorders, leukocytosis, and hypoglycemia, all phenomena that are completely resolved within 3–4 h after birth [8].

In labor context, maternal suffering is notable and is characterized by pain (labor pain) generated by uterine contractions, compression, and traction on the pelvic-genital structures [2]. Labor pain consists of a visceral component that characterizes the first and the second stage of childbirth and a somatic component that occurs during the late first stage and the second stage. However, pain perception is subjective: nulliparous pain scores are higher compared to that of multiparous woman mainly due to the lack of previous birth experience or antenatal education [17,18]. As a response to pain and labor-related stress, there is a cascade of hormones that facilitates the reduction of pain and stress levels during and after birth and stimulates the interaction and bonding between mother and baby in the postpartum period. Oxytocin is a key hormone during pregnancy in childbirth, as it contracts the uterus and promotes the progress of labor, helps the expulsion of the fetus and placenta, and prevents bleeding afterwards. Among other benefits, it reduces sympathetic nervous system activity (“fight or flight”) and increases parasympathetic nervous system activity (“relaxation and growth” and “calm and connection”), reducing anxiety and pain [19,20]. Beta-endorphin is a natural narcotic and plays an important role in pain perception modulation. Its level increases during pregnancy, reaching a peak during labor. This hormone helps women giving birth deal with pain but also supports the positive mood during childbirth process [21,22]. Catecholamines are stress hormones released during a fight-or-flight response. Their peak in the last portion of labor prior to pushing contributes towards the powerful fetal ejection reflex and help the newborn’s transition to extrauterine life [20].

 A separate remark must be made as regards the psychic component through the emotional state, depression, and stress it implies, which may interfere negatively with the evolution of pregnancy. It is estimated that the global prevalence of postpartum depression (PPD) is approximately 17%, directly associated with country development and national or regional income [23,24]. The major and moderate risk factors are prenatal depression, prenatal anxiety, a poor economic foundation, and lack of social support from partner or others [25,26,27]. Of these factors, social support has been considered both as one of the strongest predictors of PPD and a target of psychosocial intervention [28]. Several possible mechanisms associated social support to PPD. There is a strong degree of comparison with other mothers that might be a risk for PPD, whereas the support of others can relieve the stress. The incapacity of breastfeeding can also generate PPD, as mothers blame themselves and lose their confidence as mothers [29]. Challenges and difficulties of childcare are easily overpassed with encouragement from others, and this reassurance is considered a protective factor against PPD [28,30]. The lack of social support can also result in a difficult labor and birth. There is also the fear of birth, leading to higher levels of pain that may inhibit progression of labor and can exacerbate postnatal depressive symptoms [31,32]. Because of the stigma of depression, the mother may refuse to seek professional help [33]. The psychological impact that childbirth has on the mother should not be underestimated, as it may influence the evolution of the subsequent relationship with the newborn, especially since the childbirth itself is an act with a primarily neuroendocrine substrate [34]. Apart from strengthening this relationship, this impact may also have psychiatric connotations in the form of intrapartum or postpartum recurring psychosis with depressive or even schizoid manifestations. Birth is associated with a marked increase in the risk of episodes of psychiatric disorders [35,36]. Without leaving these aspects aside, one must retain the psycho-emotional and affective beneficial effect of the act of physiological birth on the relationship and subsequent evolution of the mother–child couple.

## 3. The Impact of Vaginal Birth on the Newborn

The major impact of the act of childbirth on the baby is the absolute need for immediate and subsequently gradual adaptation to a new environment of life: the extrauterine medium where the conditions are completely different and require a considerable effort of adaptation from the newborn. However, it is a complex physiological process that is verified in time and that, under normal circumstances, is concluded with the emergence of the parent–child couple [2,9]. Beyond the adaptation effort, childbirth also means exposure to a trauma that occurs along certain parameters during labor and expulsion and that at any time may become pathological, especially for primipara. The passage through the pelvic-genital channel (the birth canal) is equal to compression, flexion, and deflections of various segments of the newborn’s body, with compression and relaxation produced during the progression of birth and with effects on the organs, systems, but also nutrition of the fetus, and its most severe result, hypoxia, is a situation that must be feared due to impact on the nerve cell, blood hemostasis, and metabolic homeostasis [3,9]. From the viewpoint of the severity of trauma to which the body is subjected at birth, it is considered that under the circumstances of a normal existence, it is the most powerful and dramatic one in the individual’s life [37]. The dehiscence of the cranial sutures, laxity of tissues and their elasticity, as well as incomplete ossification make the crossing of the genital canal progressive and without major incidents, occurring almost spontaneously and without stopping in more than 95% of the cases; that is why since times immemorial the mother was assisted at birth by uneducated yet experienced people who did nothing but help nature, cutting off the cord and assisting the mother together with the newborn. They were the midwives, a profession that has emerged since antiquity and later the accoucheur, a kind of obstetrician, as we would say today [38]. The progression of birth at least as far as the fetus is concerned seems to be done in perfect silence, unlike maternal pain, agitation, and coordinated effort for expulsion. This does not mean that the newborn does not suffer or does not feel the pain.

The exact nature of fetal pain remains unknown, but there is evidence that the fetus experiences pain in the third trimester of pregnancy [39]. The perception of pain earlier during gestational period might be sustained by spino-thalamic pathways development (approximately from the 20th week) and the connections of the thalamus with the subplate (approximately from the 23rd week). The appearance of the correct neuro-anatomic connections is not sufficient to produce a mature ability to feel and interpret pain, but these developing neural elements are not inactive, as noxious stimulation can produce stress hormones very early in gestation [40]. The stress experienced by a mother during pregnancy has been correlated with later changes in children’s behavioral responses to painful stimulation. Traumatic insults can activate the fetal hypothalamic pituitary adrenal axis and negatively influence the infant’s neurodevelopment [41]. Until recently, it was thought that intrauterine inhibitory chemicals block all fetal pain throughout the pregnancy, but a literature review concluded that these neuro-inhibitors do not have an anesthetic action at normal fetal values [42].

Simply, the newborn can express pain only at the moment of his first breath, which is nothing more than a scream of pain followed by crying that most of those present consider an expression of life and vitality when it actually comes from an underlying pain accumulated throughout birth. Crucial changes, however, are the functional changes that occur with birth because the morphological changes are reduced to skull changes, as it becomes ovoid by overlapping the sutures due to the compression of the pelvic-genital passage with the risk of hemorrhage (blood diffusion, frontal bossing, cephalohematoma by periosteal accumulation of fluid, and cerebral-meningeal hemorrhages by vascular rupture due to pressure on the brain or hypoxia) [43]. The prevention of birth trauma can be achieved by using a professional multidisciplinary team for taking care of both mother and child. Advances in antenatal care can identify fetal malformations and malpresentation as potential issues, preparing the practitioner for a high-risk delivery [43]. 

However, a certain degree of perinatal stress is beneficial during childbirth, as it increases the synthesis of cortisol and catecholamine in the infant’s blood; these are hormones of great importance for the achievement of pulmonary maturity and for the adaptation of the circulatory system to extrauterine life [44,45].

Of vital importance, however, are the functional changes of the vital organs of the lung and heart that provide the vital functions: breathing and circulation, which occurs spontaneously with the sudden passage to the extrauterine life. According to Schuler et al.’s study, newborns delivered through the birth canal achieve higher cortisol levels and present an increased expression of pain compared to neonates delivered by cesarean section [37].

The lungs are still full of liquid until the first breath. The first inspiratory effort plays a critical role by generating an active pressure gradient that allows the fluid to pass into the interstitial tissue, from where it will be gradually removed by pulmonary and lymphatic circulation [6]. The first breath is the setting in motion of the respiratory apparatus, which controls the gaseous exchange with the external environment. The penetration of the air into the alveoli is accomplished mechanically by the decompression of the chest when passing through the compression of the genital canal. Simultaneously, hypoxia and hypercapnia performed by the same mechanism will set in motion the archaic bulbar and pneumotaxic centers of the pons, which will determine the respiratory rhythm. Abruptly, the child will pass from a state of chronic hypoxia and moderate respiratory acidosis to a normal state of oxygen and carbon dioxide concentration in the blood immediately followed by physiological hemolysis (neonatal jaundice) and rapid decrease of fetal hemoglobin [8,46]. The second instantaneous adaptation process is the transformation of the fetal blood circulation into adult circulation by suppressing the placental circulation (in which the nutritional intake was provided by the umbilical veins), the progressive closure of the intra-auricular communication, the triggering of the cardiopulmonary circulation (the small circulation), the closure of the arterial canal, and the completion of the portal circulation [8,47]. Childbirth also means the onset of other types of adaptation that will occur over time: renal, digestive, immunological adaptation, etc.

All these will be followed by specific care, natural nutrition (the white umbilical cord), and the psycho-affective and environmental stimulation of the mother–child couple, which will be the expression of the normal physiological evolution of vaginal birth. We emphasize that the above mentioned can be circumscribed to 95% of births [2].

## 4. Cesarean Section as a Medical Solution

Every obstetrics textbook has a chapter entitled pathology of labor, which, of course, refers to situations where, for various reasons, birth does not progress, progresses slowly, or stops evolving.

Many situations that affect birth belong to the pathology of pregnancy, which can be equally maternal, i.e., consuming diseases, basin dystocia, or congenital malformations; related to pregnancy, i.e., twins or malformations; or they may be related to the fetus, i.e., position dystocia, congenital malformations, hydrocephalus, or other situations that make it impossible or risky to give birth through the birth canal [1]. Today, the nearly exclusive solution is cesarean surgery, which has become extremely popular for use in obstetric services. The motivation for such large-scale use lies in the modern possibilities of pregnancy investigation, which makes it possible to highlight the numerous situations that could pose a fetal or maternal risk at birth; the actual desire of the parturient women to avoid the inconveniences of vaginal birth, i.e., fear of labor pain, fear of childbirth, experience of previous labor, desire to avoid long labor, anxiety due to fetal trauma/death, pelvic floor and vaginal trauma, urinary incontinence, fear of defecation, anxiety about the loss of control, anxiety due to lack of support from the staff, emotional aspects, and anomalies of the prenatal examination; and of course, the technical possibilities that offer a high degree of safety of the operation itself [48,49,50]. It seems that most of both physicians and patients’ perception about delivery methods is that spontaneous delivery is riskier than cesarean section [51]. Therefore today, at least in the urban environment, cesarean has become a routine practice. Even the history of this intervention is a historical curiosity that was perpetuated for more than 2000 years to reach its current stage of evolution.

In India and Egypt, it was practiced since the year 600 BC, and the legal regulations were instituted in the time of the Roman King Numa Pompilius in the Lex Regia, which specifically provided cesarean as a mean of extracting the child from his deceased mother. This provision could also be found in Lex Caesarea during the time of Emperor Julius Caesar somewhere between 46–44 BC, hence the name Cesarean section [42,52].

The first mention in a medical book dates back to 1350, and the first measures to apply for the parturient in difficulty belonged to Ambroise Paré in 1550 and Jeremias Trautmann in 1610, whose patient died 25 days after through septicemia. Hemorrhage, peritonitis, and septicemia were major hazards until 1882 when Sänger introduced the surgical wound suture (thus controlling hemorrhage and peritonitis caused by penetration of the lochia into the peritoneal cavity), in 1876 when Foster introduced asepsis and antisepsis, in 1847 when Simson introduced anesthesia, and in 1907 when Frank of Bonn and Hugo Selheim performed the incision on the lower uterine segment, bypassing the contractile area of the uterus and implicitly the peritoneal layer [42,53]. Modern technical possibilities have transformed cesarean into an ordinary intervention ever more demanded by the modern woman. Although maternal request is one of the main causes of increased cesarean section levels, the 2011 guidelines of cesarean section clarify that maternal request, in the absence of clinical reasons, does not specify an indication for the intervention [51,54]. The demand grew so much that while in 1970, only 5% of children were born through cesareans, in the 1980s, it was 25%, and today, it is estimated that in some centers the percentage has sometimes reached 50–75% instead of 10–15% as recommended by the World Health Organization (WHO) to be necessary in order to prevent the risks associated with vaginal births [55].

In many developed countries, the number of cesareans has increased, and attention has been focused on strategies to reduce its use, as it does not provide additional health gain but may increase maternal risks, and it may have implications for future pregnancies and health services [55,56,57,58,59,60]. The reasons for this increase are multifactorial, and they are not well understood. Changes in maternal characteristics and professional practice styles, increased malpractice pressure, as well as various economic, organizational, social, and cultural factors have been involved in this trend [61,62,63]. 

Another common reason for cesarean section is the phenomenon of defensive medicine. Defensive medicine seems to be related to gynecologists’ fear of legal actions promoted by their patients for medical malpractice and negligence [51].

Additional concerns and controversies related to cesarean section include inequalities in the use of the procedure not only between countries but also within countries as well as the costs they impose on health systems [63,64]. For Medicaid, the average costs involved in cesarean delivery were 30% higher than for vaginal deliveries, including prenatal care, childbirth, and postnatal care [65]. Usually, higher costs of cesarean delivery are explained by a higher rate of newborn admissions to neonatal intensive care units (NICU), maternal intensive care unit (ICU) admission, longer hospital stay, and greater use of human resources for assistance [4,66,67]. Perioperative complications seem to associate the highest risk for prolonged postpartum length of hospitalization after cesarean delivery, additionally increasing heath care burden [68,69]. Preterm delivery, grandmultiparity (history of ≥5 births), multiple pregnancy, the need of urgent cesarean section, abnormal placentation, and preeclampsia seem to be most frequent associated with prolonged hospital stay following cesarean delivery [70,71].

A contributor to the increased cesarean section levels might be the remuneration system that pays the same amount for vaginal and cesarean birth regardless of the prolonged assistance hours given in vaginal deliveries [72].

The highest cesarean section rates are found in Latin America and the Caribbean (40.5%), and the lowest rates are found in sub-Saharan Africa [23], indicating a lack of access to this lifesaving intervention mainly due to the paucity of resources [73,74].

It is true that modern technical possibilities also highlight many situations that could negatively influence the evolution of pregnancies that would require a cesarean section as a necessity [44].

## 5. The Morphological Implications of Cesarean Section on the Parturient

Cesarean surgery is actually a brutal interruption of cohabitation of the fetus with his mother through the surgical separation that this procedure implies. It can be said that although the intervention is scheduled, and the psychological preparation of the mother is fulfilled, physiologically, neither the maternal nor the fetus’s body is prepared [69]. It means, in other words, transforming the physiological event of childbirth into an artificial, forced one and that both mother and infant will bear the impact of the intervention involving the surgical procedure, the anesthesia, the absence of labor and expulsion, and the entire neurohormonal and functional sequence that birth involves as well as the risk of trauma by traction and by the use of instruments, the risk of bleeding, and the risk of infections [75].

WHO’s recommendation of initiating breastfeeding is within one hour of birth [76]. Although skin-to-skin and early breastfeeding should always be the routine after birth regardless of mode of birth, in cesarean section delivery, most probably, the mother will not touch and breastfeed the baby until after a variable time of at least 24 h, with the mother being under anesthesia, and lactation due to the severity of separation and the lack of hormonal impact will be delayed [77,78,79,80,81]. 

As for the number of cesarean sections a woman can have, research has not established an exact number of repeat cesarean sections considered safe. Compared with primary caesarian section, multiple repeat caesarean sections (MRCS) are associated with additional risks including placenta previa, abnormal placental invasion, and difficulties in surgical dissection [43,82]. Furthermore, maternal morbidity increases with increasing numbers of previous cesarean sections [83] (Table 1).

## 6. The Impact of Cesarean on the Fetus

This impact is not less important because the same intervals that physiological birth implies are bypassed through cesarean. The slow adaptation to the new living environment involved by the passage through the pelvic-genital canal is brutally interrupted. The first breath is no longer the result of decompression of the thorax that passed through the pelvic strain but the result of artificial skin stimulation, thoracic massage, or ventilation with the balloon. The presence of the anesthetic in the fetal circulation diminishes the tone and reflexes of the newborn, delaying the immediate adaptation reaction.

It is known that the newborn needs >5 min to attain an arterial oxygen saturation >80% and almost 10 min to reach 90% during a normal postnatal transition [89]. Cesarean section is usually associated with either general or regional anesthesia, both with high impact on the newborn’s adaptation to extrauterine environment. Regional anesthesia is frequently the first choice for elective cesarean. General anesthesia or conversion from regional to general anesthesia is required for emergency reasons. Several studies identified significant differences in newborn outcomes between general and regional anesthesia [90]. General anesthesia is associated with lower APGAR score in the 1st, 3rd, and 5th minute following cesarean delivery compared to regional anesthesia [90,91,92]. Sung et al. compared the effect of general and spinal anesthesia for elective cesarean section. The authors found a larger proportion of newborns with 5-minute Apgar scores < 7 in the general anesthesia group than in the spinal group [93]. Similarly, Bao et al. reported significant differences in newborn outcomes between the general and neuro-axial anesthesia groups concerning APGAR score in the 1st and 5th minute. Admission rate to Neonatal Intensive Care Unit (NICU) was greater in the general anesthesia group [91]. 

Knigin et al. evaluated the effect of maternal hypotension after spinal anesthesia for elective cesarean and the time from anesthesia to delivery as risk factors for neonatal acidosis. Anesthesia-to-incision and incision-to-delivery intervals, use of vasopressor treatment, and sustained spinal hypotension were independently associated with neonatal acidosis. However, no neonatal complications such as transient tachypnea of the newborn, respiratory distress, or admission to NICU were encountered. Other factors such as maternal age, the number of previous cesarean deliveries, gestational age, neonatal birthweight, and fetal presentation were not associated with neonatal acidosis [94]. 

In terms of newborn cerebral oxygen saturation measured immediately after cesarean delivery, Willfurth et al. concluded that cerebral tissue oxygenation in neonates during immediate transition after birth was similar after maternal general and spinal anesthesia despite differences in SpO2, heart rate (HR), and APGAR score in term neonates. In preterm neonates, there were no statistically significant differences in SpO2, HR, and cerebral oxygen saturation between the two groups of study. Only the APGAR score in the 1st minute was significant lower in the general anesthesia group [95]. In contrast, Ozgen et al. found cerebral oxygen saturation to be higher in the combined spinal epidural anesthesia group than in the general anesthesia group. As for SpO2 values, the neonates delivered by cesarean section performed under combined spinal epidural anesthesia had higher SpO2 values than neonates born from mothers given general anesthesia [96]. Urlesberger et al. analyzed the value of cerebral oxygen saturation measured in term infants born via vaginal birth versus cesarean delivery but found no difference between the groups. SpO2 and HR levels were lower during the observational period in the cesarean section group [97].

The absence of positive stimuli determined by the mother–child relationship that is delayed in cesarean delivery would negatively influence the child’s adaptation, especially because nutrition is delayed by the absence of lactation or the impossibility to practice it during the first 24 h [1,2,8]. The forced expulsion of the fetus causes sudden decompression of the head as well as possible intracranial hemorrhage [98]. However, many speculations are based on this adaptation to the extrauterine life of a cesarean-born baby, such as thermoregulation, hormonal activity, the enzyme behavior of the new-born baby, the reaction to the environment, and immune response. However, these adaptations are characteristic of the neonatal period and childhood; they occur over time, and their maturation follows a route that does not depend on the type of birth; the digestive adaptation is accomplished in the first year and immunological maturity at 10 years, not to mention the morpho-functional maturity, which is complete in adolescence. 

Regarding the pediatric consequences of cesarean section, several studies concluded that cesarean section is a risk factor for respiratory tract infections, asthma, obesity, and neurological disorders in children [45]. Current studies consider early alteration of the human microbiome to play an important role in the onset and progression of several diseases by modulating important metabolic and immunomodulatory processes [99,100]. Depending on the delivery mode, quantitative and qualitative differences have been found in newborns’ intestinal microflora [45]. Infants born through the birth canal share their mothers’ vaginal and fecal flora, while those delivered through cesarean section have a microbiota similar to their mothers’ skin and to the surrounding environment [100]. 

During the last years, the relationship between cesarean section and childhood asthma has been a subject of debate, with contradictory opinions available in the literature. Darabi et al., in their systematic review and meta-analysis, concluded that cesarean section, whether elective or performed for emergency reasons, increased the risk of childhood asthma [101]. The lack of contact with maternal vaginal microbiota, which is necessary for the growth and development of the newborn’s immune system and the completion of pregnancy without perinatal stress hormone responsible for lung maturation, both increased the risk of difficulties in extrauterine life adaptation and long-term negative effects on lung function [102]. Cesarean birth influences the risk of asthma partially by gut microbial colonization and perturbed immune responses reflected by dysregulations in bile acid and tryptophan metabolism during early life [103]. In their study on cesarean section without medical indication and the risk of childhood allergic disorder, Chu et al. reported that the risk of childhood asthma might be attenuated by breastfeeding although further research is required [104].

Lavin et al. compared the risk of obesity in childhood of children born through cesarean section (elective and performed in emergency) and children born through vaginal birth. Their findings suggested that there might be an association between cesarean section and childhood overweight [105]. Concerning the risk of childhood obesity following cesarean delivery, Zhang et al. recently realized the first systematic review and meta-analysis focused on the association between elective cesarean and children’s weight development as long-term outcome. Their study concluded that children delivered through cesarean section present an increased risk of obesity from infancy to adolescence [106]. Obesity and insulin resistance may come as a result of the chronic inflammation produced by an altered intestinal microbiota and its metabolites [107].

Cesarean section is thought to have a negative effect on early brain development. Zhang et al. conducted a systematic review and meta-analysis to evaluate neurodevelopment and psychiatric pathologies in children delivered via cesarean section compared to those born through vaginal birth. They concluded that cesarean section was associated with an increased risk of several disorders. Autism spectrum disorder (ASD) and attention deficit/hyperactivity disorder (ADHD) were most statistically significant [108]. Similarly, Zhang et al. reported that children born via planned or intrapartum cesarean section experienced an increased risk of neurodevelopmental disorders such as ADHD and intellectual disability compared to children born through vaginal delivery. These findings were mainly explained by familial factors [109]. According to Blazkova et al., the mode of delivery seems to have a significant influence in psychological cognitive tests applied on 5-year-old children, with cesarean-born children obtaining notably lower scores than vaginally born children [110]. Regarding the IQ score of children delivered by cesarean section and those delivered vaginally through the birth canal, Khadem et al. observed no significant difference in IQ scores of children born vaginally with respect to those delivered by cesarean section [111]. It is thought that a normal human microbiome is essential for central nervous system development and emotional regulation. Any imbalance of the normal intestinal microbiota, such as the one resulting in the newborn’s microbiota after cesarean section delivery, affects the central nervous system under the action of the microbe–gut–brain axis through nerve, immune, endocrine, and metabolic pathways, increasing the risk of neuropsychiatric disorders [112]. However, the association between cesarean section and behavioral pathology needs additional evidence, and it is a direction of future research.

## 7. Conclusions

Birth is a physiological act that is part of the morpho-functional economy of the maternal body. Each stage in the act of birth has a predetermined pathway that is neurohormonally induced and morpho-functionally established through specific and characteristic adaptations. Like maternity, childbirth also has an important impact on the maternal body as a biological structure and psycho-emotional behavior as well as on her social life and relationships.

The passage through the pelvic-genital canal in the act of childbirth is accompanied by important functional processes with a decisive role in adapting the newborn to extrauterine life, with beneficial effects on both the mother and child. Mother–child cohabitation and breastfeeding can be restored after the physiological birth.

Vaginal birth remains the path of choice for a physiological evolution of pregnancy. Although erroneously considered safe and easy today, cesarean section delivery must remain an emergency procedure or a procedure recommended for pregnancies where birth is a risk to the mother and to the child.

## Figures and Tables

**Table 1:** Cesarean section associated risks.

	Associated Risk		Reference
Mother	Intraoperative	Anesthesia-associated risks	[84]
		Infections	[69,75]
		Organ injury (bladder, bowel, ureter, etc.)	[75]
		Need for blood transfusion or hysterectomy	[84]
	Postoperative	Thromboembolic complications (embolism, thrombosis)	[84]
		Pelvic adhesions	[85]
		Persistent pain	[84]
		Delayed initiation of breastfeeding	[80]
Newborn	Difficult adaptation to extrauterine life		[69,75]
	Altered immune system development		[75]
	Increased risk of developing asthma and allergies		[86]
	Reduced gut microbiome diversity		[87]
Future pregnancy	Intrauterine growth restriction		[69]
	Ectopic pregnancy		[75]
	Preterm birth		[75]
	Stillbirth		[75]
	Spontaneous abortion		[84]
	Uterine rupture		[85]
	Abnormal placentation (placenta praevia, increta, or accreta) with possbile bleeding and need for blood transfusion and hysterectomy		[85]
	Infertility		[84,87,88]

## Data Availability

Data sharing not applicable.

## References

[1] Aburel E. (1971). Obsterică și Ginecologie.

[2] Lupu V. (1996). Știința Creșterii și Dezvoltării Copilului.

[3] Technical Working Group, World Health Organization (1997). Care in normal birth: A practical guide. Birth.

[4] Negrini R., da Silva Ferreira R.D., Guimarães D.Z. (2021). Value-based care in obstetrics: Comparison between vaginal birth and caesarean section. BMC Pregnancy Childbirth.

[5] Bakke E., Øseth E.H., Fofanah T., Sesay I., van Duinen A., Bolkan H.A., Westendorp J., Lonnee-Hoffmann R. (2022). Vacuum births and barriers to its use: An observational study in governmental hospitals in Sierra Leone. BMJ. Open.

[6] Stelzer I.A., Ghaemi M.S., Han X., Ando K., Hédou J.J., Feyaerts D., Peterson L.S., Rumer K.K., Tsai E.S., Ganio E.A. (2021). Integrated trajectories of the maternal metabolome, proteome, and immunome predict labor onset. Sci. Transl. Med..

[7] Vannuccini S., Bocchi C., Severi F.M., Challis J.R., Petraglia F. (2016). Endocrinology of human parturition. Ann Endocrinol..

[8] Trifan N.N. (1982). Pediatrie Preventive.

[9] Constantinescu C., Petrescu C. (1968). Puericultură și Pediatrie.

[10] Shynlova O., Lee Y.H., Srikhajon K., Lye S.J. (2013). Physiologic uterine inflammation and labor onset: Integration of endocrine and mechanical signals. Reprod. Sci..

[11] Abalos E., Oladapo O.T., Chamillard M., Díaz V., Pasquale J., Bonet M., Souza J.P., Gülmezoglu A.M. (2018). Duration of spontaneous labour in ‘low-risk’ women with ‘normal’ perinatal outcomes: A systematic review. Eur. J. Obstet. Gynecol. Reprod. Biol..

[12] Caughey A.B. (2020). Is Zhang the new Friedman: How should we evaluate the first stage of labor?. Semin. Perinatol..

[13] Management of the Third Stage of Labor. https://emedicine.medscape.com/article/275304-overview.

[14] Normal Labour and Delivery. https://emedicine.medscape.com/article/260036-overview#a3.

[15] Ouzounian J.G., Elkayam U. (2012). Physiologic changes during normal pregnancy and delivery. Cardiol. Clin..

[16] Hesson K., Hill T., Bakal D. (1997). Variability in breathing patterns during latent labor. A pilot study. J. Nurse Midwifery.

[17] Labor S., Maguire S. (2008). The Pain of Labour. Rev Pain.

[18] Vanderlaan J., Sadler C., Kjerulff K. (2021). Association of Delivery Outcomes with the Number of Childbirth Education Sessions. J. Perinat Neonatal Nurs..

[19] Uvnäs-Moberg K., Ekström-Bergström A., Berg M., Buckley S., Pajalic Z., Hadjigeorgiou E., Kotłowska A., Lengler L., Kielbratowska B., Leon-Larios F. (2019). Maternal plasma levels of oxytocin during physiological childbirth—A systematic review with implications for uterine contractions and central actions of oxytocin. BMC Pregnancy Childbirth.

[20] Olza I., Uvnas-Moberg K., Ekström-Bergström A., Leahy-Warren P., Karlsdottir S.I., Nieuwenhuijze M., Villarmea S., Hadjigeorgiou E., Kazmierczak M., Spyridou A. (2020). Birth as a neuro-psycho-social event: An integrative model of maternal experiences and their relation to neurohormonal events during childbirth. PLoS ONE.

[21] Walter M.H., Abele H., Plappert C.F. (2021). The Role of Oxytocin and the Effect of Stress During Childbirth: Neurobiological Basics and Implications for Mother and Child. Front. Endocrinol..

[22] Altınayak S.Ö., Özkan H. (2022). The effects of conventional, warm and cold acupressure on the pain perceptions and beta-endorphin plasma levels of primiparous women in labor: A randomized controlled trial. Explore.

[23] Wisner K.L., Sit D.K.Y., McShea M.C., Rizzo D.M., Zoretich R.A., Hughes C.L., Eng H.F., Luther J.F., Wisniewski S., Costantino M.L. (2013). Onset Timing, Thoughts of Self-harm, and Diagnoses in Postpartum Women With Screen-Positive Depression Findings. JAMA Psychiatry.

[24] Ferrari B., Mesiano L., Benacchio L., Ciulli B., Donolato A., Riolo R. (2021). Prevalence and risk factors of postpartum depression and adjustment disorder during puerperium—A retrospective research. J. Reprod. Infant Psychol..

[25] Qi W., Zhao F., Liu Y., Li Q., Hu J. (2021). Psychosocial risk factors for postpartum depression in Chinese women: A meta-analysis. BMC Pregnancy Childbirth.

[26] Waller R., Kornfield S.L., White L.K., Chaiyachati B.H., Barzilay R., Njoroge W., Parish-Morris J., Duncan A., Himes M.M., Rodriguez Y. (2022). Clinician-reported childbirth outcomes, patient-reported childbirth trauma, and risk for postpartum depression. Arch. Womens Ment. Health.

[27] Khsim I.E.F., Rodríguez M.M., Riquelme Gallego B., Caparros-Gonzalez R.A., Amezcua-Prieto C. (2022). Risk Factors for Post-Traumatic Stress Disorder after Childbirth: A Systematic Review. Diagnostics.

[28] Yamada A., Isumi A., Fujiwara T. (2020). Association between Lack of Social Support from Partner or Others and Postpartum Depression among Japanese Mothers: A Population-Based Cross-Sectional Study. Int. J. Environ. Res. Public Health.

[29] Berman Z., Thiel F., Dishy G.A., Chan S.J., Dekel S. (2021). Maternal psychological growth following childbirth. Arch. Womens Ment. Health.

[30] Tasuji T., Reese E., van Mulukom V., Whitehouse H. (2020). Band of mothers: Childbirth as a female bonding experience. PLoS ONE.

[31] Hulsbosch L.P., Boekhorst M.G.B.M., Potharst E.S., Pop V.J.M., Nyklíček I. (2021). Trait mindfulness during pregnancy and perception of childbirth. Arch. Womens Ment. Health.

[32] Deng Y., Lin Y., Yang L., Liang Q., Fu B., Li H., Zhang H., Liu Y. (2021). A comparison of maternal fear of childbirth, labor pain intensity and intrapartum analgesic consumption between primiparas and multiparas: A cross-sectional study. Int. J. Nurs. Sci..

[33] Zeleke T.A., Getinet W., Tadesse Tessema Z., Gebeyehu K. (2021). Prevalence and associated factors of post-partum depression in Ethiopia. A systematic review and meta-analysis. PLoS ONE.

[34] Shakarami A., Mirghafourvand M., Abdolalipour S., Jafarabadi M.A., Iravani M. (2021). Comparison of fear, anxiety and self-efficacy of childbirth among primiparous and multiparous women. BMC Pregnancy Childbirth.

[35] Munk-Olsen T., Laursen T.M., Pedersen C.B., Mors O., Mortensen P.B. (2006). New Parents and Mental Disorders: A Population-Based Register Study. JAMA.

[36] Harlow B.L., Vitonis A.F., Sparén P., Cnattingius S., Joffe H., Hultman C.M. (2007). Incidence of hospitalization for postpartum psychotic and bipolar episodes in women with and without prior prepregnancy or prenatal psychiatric hospitalizations. Arch. Gen Psychiatry.

[37] Schuller C., Känel N., Müller O., Kind A.B., Tinner E.M., Hösli I., Zimmermann R., Surbek D. (2012). Stress and pain response of neonates after spontaneous birth and vacuum-assisted and cesarean delivery. Am. J. Obst. Gynecol..

[38] Iftimovici R. (2015). Istoria universală a medicinei și farmaciei.

[39] Bellieni C.V., Buonocore G. (2012). Is fetal pain a real evidence?. J. Matern.-Fetal Neonatal Med..

[40] Pierucci R. (2020). Fetal Pain: The Science Behind Why It Is the Medical Standard of Care. Linacre Q..

[41] Sanders M.R., Hall S. (2017). L Trauma-informed Care in the Newborn Intensive Care Unit: Promoting Safety, Security and Connectedness. J. Perinatol..

[42] Bellieni Carlo V., Vannuccini Silvia V., Petraglia Felice V. (2018). Is Fetal Analgesia Necessary during Prenatal Surgery?. J. Matern -Fetal Neonatal Med..

[43] Zwergel C., Kaisenberg C. (2019). Maternal and Fetal Risks in Higher Multiple Cesarean Deliveries. Recent Advances in Cesarean Delivery.

[44] Dumpa V., Kamity R. (2022). Birth Trauma. StatPearls.

[45] Słabuszewska-Jóźwiak A., Szymański J.K., Ciebiera M., Sarecka-Hujar B., Jakiel G. (2020). Pediatrics Consequences of Caesarean Section-A Systematic Review and Meta-Analysis. Int. J. Environ. Res. Public Health.

[46] LoMauro A., Aliverti A. (2016). Physiology masterclass: Extremes of age: Newborn and infancy. Breathe.

[47] Friedman A.H., Fahley J.T. (1993). The transition from fetal to neonatal circulation: Normal responses and implications for infants with heart disease. Semin. Perinatol..

[48] Wigert H., Nilsson C., Dencker A., Begley C., Jangsten E., Sparud-Lundin C., Mollberg M., Patel H. (2020). Women’s experiences of fear of childbirth: A metasynthesis of qualitative studies. Int. J. Qual.Stud. Health Well-Being.

[49] Miller Y.D., Danoy-Monet M. (2021). Reproducing fear: The effect of birth stories on nulligravid women’s birth preferences. BMC Pregnancy Childbirth.

[50] Coates D., Thirukumar P., Spear V., Brown G., Henry A. (2020). What are women’s mode of birth preferences and why? A systematic scoping review. Women Birth.

[51] Fineschi V., Arcangeli M., Di Fazio N., Del Fante Z., Fineschi B., Santoro P., Frati P. (2021). Defensive Medicine in the Management of Cesarean Delivery: A Survey among Italian Physicians. Healthcare.

[52] Appendix: Creative Etymology: “Caesarean Section” from Pliny to Rousset. https://www.jstor.org/stable/10.7591/j.ctvn1tb31.9.

[53] Boley J.P. (1935). The history of caesarean section. Can. Med. Assoc. J..

[54] National Institute for Health and Clinical Excellence Cesarean Section. https://www.nice.org.uk/guidance/cg132/documents/cesarean-section-update-full-guideline2.

[55] World Health Organization (1985). Appropriate technology for birth. Lancet.

[56] Thomas J., Paranjothy S. (2001). Royal College of Obstetricians and Gynaecologists Clinical Effectiveness Support Unit.

[57] National Collaborating Centre for Women’s and Children’s Health (2004). Caesarean Section: Clinical Guideline.

[58] Timor-Tritsch I.E., Monteagudo A. (2012). Unforeseen consequences of the increasing rate of cesarean deliveries: Early placenta accreta and cesarean scar pregnancy. A review. Am. J. Obstet. Gynecol..

[59] Gregory K.D., Jackson S., Korst L., Fridman M. (2012). Cesarean versus vaginal delivery: Whose risks? Whose benefits?. Am. J. Perinatol..

[60] Opiyo N., Kingdon C., Oladapo O.T., Souza J.P., Vogel J.P., Bonet M., Bucagu M., Portela A., McConville F., Downe S. (2020). Non-clinical interventions to reduce unnecessary caesarean sections: WHO recommendations. Bull. World Health Organ..

[61] Zwecker P., Azoulay L., Abenhaim H.A. (2011). Effect of fear of litigation on obstetric care: A nationwide analysis on obstetric practice. Am. J. Perinatol..

[62] Mi J., Liu F. (2014). Rate of caesarean section is alarming in China. Lancet.

[63] Gibbons L., Belizan J.M., Lauer J.A., Betran A.P., Merialdi M., Althabe F. (2012). Inequities in the use of cesarean section deliveries in the world. Am. J. Obstet. Gynecol..

[64] Ronsmans C., Holtz S., Stanton C. (2006). Socioeconomic differentials in caesarean rates in developing countries: A retrospective analysis. Lancet.

[65] Truven Health Analytics (2013). The Cost of Having a Baby in the United States. http://transform.childbirthconnection.org/wp-content/uploads/2013/01/Cost-of-Having-aBaby-Executive-Summary.pdf.

[66] MacLellan A.N., Woolcott C.G., Brown M.M., Dodds L., McDonald S.D., Kuhle S. (2019). Cesarean Delivery and Healthcare Utilization and Costs in the Offspring: A Retrospective Cohort Study. J. Pediatr..

[67] Etringer A.P., Pinto M.F.T., Gomes M.A.S.M. (2019). Análise de custos da atenção hospitalar ao parto vaginal e à cesariana eletiva para gestantes de risco habitual no Sistema Único de Saúde. Cien Saude Colet.

[68] Blumenfeld Y.J., El-Sayed Y.Y., Lyell D.J., Nelson L.M., Butwick A.J. (2015). Risk Factors for Prolonged Postpartum Length of Stay Following Cesarean Delivery. Am. J. Perinatol..

[69] Sobhy S., Arroyo-Manzano D., Murugesu N., Karthikeyan G., Kumar V., Kaur I., Fernandez E., Gundabattula S.R., Betran A.P., Khan K. (2019). Maternal and perinatal mortality and complications associated with caesarean section in low-income and middle-income countries: A systematic review and meta-analysis. Lancet.

[70] Teigen N.C., Sahasrabudhe N., Doulaveris G., Xie X., Negassa A., Bernstein J., Bernstein P.S. (2020). Enhanced recovery after surgery at cesarean delivery to reduce postoperative length of stay: A randomized controlled trial. Am. J. Obstet. Gynecol..

[71] Gabbai D., Attali E., Ram S., Amikam U., Ashwal E., Hiersch L., Gamzu R., Yogev Y. (2022). Prediction model for prolonged hospitalization following cesarean delivery. Eur. J. Obstet. Gynecol. Reprod. Biol..

[72] Vogt S.E., Silva K.S., Dias M.A.B. (2014). Comparison of childbirth care models in publicmhospitals, Brazil. Rev. Saude Publica.

[73] Betran A.P., Ye J., Moller A.B., Souza J.P., Zhang J. (2021). Trends and projections of caesarean section rates: Global and regional estimates. BMJ. Glob. Health.

[74] Caesarean Section Rates Continue to Rise, Amid Growing Inequalities in Access. https://www.who.int/news/item/16-06-2021-caesarean-section-rates-continue-to-rise-amid-growing-inequalities-in-access.

[75] Sandall J., Tribe R.M., Avery L., Mola G., Visser G.H., Homer C.S., Gibbons D., Kelly N.M., Kennedy H.P., Kidanto H. (2018). Short-term and long-term effects of caesarean section on the health of women and children. Lancet.

[76] Breastfeeding. https://www.who.int/news-room/facts-in-pictures/detail/breastfeeding.

[77] WHO Global Strategy for Infant and Young Child Feeding. http://apps.who.int/iris/bitstream/10665/42590/1/9241562218.pdf?ua=1&ua=1.

[78] UNICEF, WHO (2018). Capture the Moment: Early Initiation of Breastfeeding: The Best Start for Every Newborn.

[79] Karaahmet A.Y., Bilgiç F.Ş. (2022). Breastfeeding success in the first 6 months of online breastfeeding counseling after cesarean delivery and its effect on anthropometric measurements of the baby: A randomized controlled study. Rev. Assoc. Med. Bras..

[80] Hobbs A.J., Mannion C.A., McDonald S.W., Brockway M., Tough S.C. (2016). The impact of caesarean section on breastfeeding initiation, duration and difficulties in the first four months postpartum. BMC Pregnancy Childbirth.

[81] Juan J., Zhang X., Wang X., Liu J., Cao Y., Tan L., Gao Y., Qiu Y., Yang H. (2022). Association between Skin-to-Skin Contact Duration after Caesarean Section and Breastfeeding Outcomes. Children.

[82] Biler A., Ekin A., Ozcan A., Inan A.H., Vural T., Toz E. (2017). Is it safe to have multiple repeat cesarean sections? A high volume tertiary care center experience. Pak. J. Med. Sci..

[83] Narava S., Pokhriyal S.C., Singh S.B., Barpanda S., Bricker L. (2020). Outcome of multiple cesarean sections in a tertiary maternity hospital in the United Arab Emirates: A retrospective analysis. Eur. J. Obstet. Gynecol. Reprod. Biol..

[84] Keag O.E., Norman J.E., Stock S.J. (2018). Long-term risks and benefits associated with cesarean delivery for mother, baby, and subsequent pregnancies: Systematic review and meta-analysis. PLoS Med..

[85] Antoine C., Young B.K. (2020). Cesarean section one hundred years 1920–2020: The Good, the Bad and the Ugly. J. Perinat. Med..

[86] Gu L., Zhang W., Yang W., Liu H. (2019). Systematic review and meta-analysis of whether cesarean section contributes to the incidence of allergic diseases in children: A protocol for systematic review and meta-analysis. Medicine.

[87] Hoang D.M., Levy E.I., Vandenplas Y. (2021). The impact of Caesarean section on the infant gut microbiome. Acta Paediatr..

[88] Nobuta Y., Tsuji S., Kitazawa J., Hanada T., Nakamura A., Zen R., Amano T., Murakami T. (2022). Decreased Fertility in Women with Cesarean Scar Syndrome Is Associated with Chronic Inflammation in the Uterine Cavity. Tohoku J. Exp Med..

[89] Kamlin C.O., O’Donnell C.P., Davis P.G., Morley C.J. (2006). Oxygen saturation in healthy infants immediately after birth. J. Pediatr..

[90] Iddrisu M., Khan Z.H. (2021). Anesthesia for cesarean delivery: General or regional anesthesia—A systematic review. Ain-Shams J. Anesthesiol..

[91] Bao Y., Zhang T., Li L., Zhou C., Liang M., Zhou J., Wang C. (2022). A retrospective analysis of maternal complications and newborn outcomes of general anesthesia for cesarean delivery in a single tertiary hospital in China. BMC Anesthesiol..

[92] Metogo J.A.M., Nana T.N., Ngongheh B.A., Nyuydzefon E.B., Adjahoung C.A., Tochie J.N., Minkande J.Z. (2021). General versus regional anaesthesia for caesarean section indicated for acute foetal distress: A retrospective cohort study. BMC Anesthesiol..

[93] Sung T.Y., Jee Y.S., You H.J., Cho C.K. (2021). Comparison of the effect of general and spinal anesthesia for elective cesarean section on maternal and fetal outcomes: A retrospective cohort study. Anesth. Pain Med..

[94] Knigin D., Avidan A., Weiniger C.F. (2020). The effect of spinal hypotension and anesthesia-to-delivery time interval on neonatal outcomes in planned cesarean delivery. Am. J. Obstet. Gynecol..

[95] Willfurth I., Baik-Schneditz N., Schwaberger B., Mileder L., Schober L., Urlesberger B., Pichler G. (2019). Cerebral Oxygenation in Neonates Immediately after Cesarean Section and Mode of Maternal Anesthesia. Neonatology.

[96] Ozgen Z.S.U., Toraman F., Erkek E., Sungur T., Guclu P., Durmaz S., Bilgili C.O. (2014). Cesarean under general or epidural anesthesia: Does it differ in terms of regional cerebral oxygenation?. Acta Anaesthesiol. Taiwan.

[97] Urlesberger B., Kratky E., Rehak T., Pocivalnik M., Avian A., Czihak J., Müller W., Pichler G. (2011). Regional oxygen saturation of the brain during birth transition of term infants: Comparison between elective cesarean and vaginal deliveries. J. Pediatr..

[98] Symonds I., Arulkumaran S., Livingstone C. (2013). Essential Obstetrics Gynaecology.

[99] Hajjo R., Sabbah D.A., Al Bawab A.Q. (2022). Unlocking the Potential of the Human Microbiome for Identifying Disease Diagnostic Biomarkers. Diagnostics.

[100] Bozomitu L., Miron I., Adam Raileanu A., Lupu A., Paduraru G., Marcu F.M., Buga A.M.L., Rusu D.C., Dragan F., Lupu V.V. (2022). The Gut Microbiome and Its Implication in the Mucosal Digestive Disorders. Biomedicines.

[101] Darabi B., Rahmati S., HafeziAhmadi M.R., Badfar G., Azami M. (2019). The association between caesarean section and childhood asthma: An updated systematic review and meta-analysis. Allergy Asthma Clin. Immunol..

[102] Liu A.H. (2015). Revisiting the hygiene hypothesis for allergy and asthma. J. Allergy Clin. Immunol..

[103] Gürdeniz G., Ernst M., Rago D., Kim M., Courraud J., Stokholm J., Bønnelykke K., Björkbom A., Trivedi U., Sørensen S.J. (2022). Neonatal metabolome of caesarean section and risk of childhood asthma. Eur. Resp. J..

[104] Chu S., Zhang Y., Jiang Y., Sun W., Zhu Q., Wang B., Jiang F., Zhang J. (2017). Cesarean section without medical indication and risks of childhood allergic disorder, attenuated by breastfeeding. Sci. Rep..

[105] Lavin T., Preen D.B. (2018). Investigating Caesarean Section Birth as a Risk Factor for Childhood Overweight. Child Obes..

[106] Zhang S., Qin X., Li P., Huang K. (2022). Effect of Elective Cesarean Section on Children’s Obesity From Birth to Adolescence: A Systematic Review and Meta-Analysis. Front. Pediatr..

[107] Ficara M., Pietrella E., Spada C., Muttini E.D.C., Lucaccioni L., Iughetti L., Berardi A. (2020). Changes of intestinal microbiota in early life. J. Matern.-Fetal Neonatal Med..

[108] Zhang T., Sidorchuk A., Sevilla-Cermeño L., Vilaplana-Pérez A., Chang Z., Larsson H., Mataix-Cols D., de la Cruz L.F. (2019). Association of Cesarean Delivery With Risk of Neurodevelopmental and Psychiatric Disorders in the Offspring: A Systematic Review and Meta-analysis. JAMA Netw. Open.

[109] Zhang T., Brander G., Mantel Ä., Kuja-Halkola R., Stephansson O., Chang Z., Larsson H., Mataix-Cols D., de la Cruz L.F. (2021). Assessment of Cesarean Delivery and Neurodevelopmental and Psychiatric Disorders in the Children of a Population-Based Swedish Birth Cohort. JAMA Netw. Open.

[110] Blazkova B., Pastorkova A., Solansky I., Veleminsky M., Veleminsky M., Rossnerova A., Honkova K., Rossner P., Sram R. (2020). The Impact of Cesarean and Vaginal Delivery on Results of Psychological Cognitive Test in 5 Year Old Children. Medicina.

[111] Khadem N., Khadivzadeh T. (2010). The intelligence quotient of school aged children delivered by cesarean section and vaginal delivery. Iran. J. Nurs. Midwifery Res..

[112] Yang H., Liu Y., Cai R., Li Y., Gu B. (2021). A narrative review of relationship between gut microbiota and neuropsychiatric disorders: Mechanisms and clinical application of probiotics and prebiotics. Ann. Palliat. Med..

